# The Great Vasculitis Pretenders: Mycotic Pseudoaneurysm, Aortitis with Occlusive Iliac Thrombus, and Paraneoplastic Aortitis. A Case-Based Review

**DOI:** 10.31138/mjr.220125.era

**Published:** 2025-08-20

**Authors:** Cristine Kuzhuppilly Arcilla, Tomas Marek, Gurjit Kaeley

**Affiliations:** 1 Department of Rheumatology, University of Florida College of Medicine, Jacksonville, Florida, USA;; 2 Department of Radiology, University of Florida College of Medicine, Jacksonville, Florida, USA

**Keywords:** infectious aortitis, paraneoplastic aortitis, mortality and complications, immunosuppression, vasculitis complications, aortitis aetiology

## Abstract

Vasculitides encompass a group of inflammatory diseases that affect blood vessels, leading to vessel wall thickening, ischemia, and potential organ damage. While rare, the prevalence of vasculitis has increased in recent years. Its presentation often mimics infectious and paraneoplastic diseases, requiring the identification of risk factors and individualised diagnostic workup. Delayed or inaccurate diagnosis of vasculitis can result in significant morbidity, vascular complications, and death since specific therapy depends on underlying aetiology and pathology. This case series highlights three patients presenting with mycotic pseudoaneurysm, infectious occlusive iliac thrombus relative to a chronic state of immunosuppression, infections, and malignancy associated with Crohn-related fistulas, *Campylobacter fetus* bacteraemia, and paraneoplastic aetiologies, resulting in poor patient outcomes. It is crucial to distinguish primary vasculitis from secondary aetiologies and mimickers, as misdiagnosis can lead to unnecessary, potentially harmful treatments and delay in surgical interventions if indicated.

## INTRODUCTION

Vasculitides are a set of rheumatologic diseases associated with inflammation of blood vessels causing intimal hyperplasia, wall thickening, oedema, and subsequent occlusion, ischemia, and necrosis to vessel walls affecting multiple organs.^1, 2^ It is rare, but its prevalence and incidence have increased over the past few years.^3^ The role of infectious pathogens affecting all vessel sizes with similar vasculitis-like presentations has been widely reported.^4–6^ Several mechanisms have been theorised for infectious vasculitis including colonisation of the aorta through vasa vasorum especially with existing pathologic damages in the vessel wall secondary to trauma, atherosclerotic plaques, or congenital or preexisting malformations. Direct endothelial invasion and necrosis were observed in Staphylococcus aureus and rickettsia, Pseudomonas aeruginosa, Legionella pneumophila, and Fusobacterium necrophorum.^4–5^ Contiguous or hematologic spread, septicaemia, and septic embolisation causing mycotic aneurysms were the pathogenic mechanisms for Staphylococcus, Streptococcus, and Salmonella.^4^ Hepatitis B and C viruses are more immune complex-mediated with subsequent complement activation.^4–5^ At the same time, other pathogens demonstrate autoreactive humoral and cell-mediated activation as part of indirect invasion.^4^ Studies reported associations with respiratory tract infection to the onset of biopsy-confirmed giant cell arteries (GCA), including parainfluenza and influenza.^4,7^ Proposed pathomechanisms are related to dendritic cell activation triggering chemotactic factors for T cell and macrophage activation, forming granulomatous infiltrates through the vasa vasorum.^7^ Other studies reported group A beta-haemolytic streptococcus cases in children associated with Henoch-Schönlein purpura and mycobacterium tuberculosis presenting as Takayasu’s arteritis in highly endemic areas.^4^ Other more established associations for certain vasculitis include Hepatitis B virus (HBV) for polyarteritis nodosa (PAN), and Hepatitis C for mixed cryoglobulinaemic vasculitis are well described.^8^ Rare cases of HIV-related vasculitis presenting with skin lesions, such as digital ischemia and leukocytoclastic vasculitis, were also identified.^9^ Most common areas for infectious vasculitic predisposition usually involve the aorta, intracranial, superior mesenteric, and femoral arteries.^10^ Vascular complications, progressive disease course, and aortic aneurysm-related deaths have been reported as part of the clinical course of aortitis, particularly with a delay in accurate diagnosis and initiation of immediate and life-saving standard-of-care interventions.^11^ Mortality is reported to be as high as 30–50% with thoracic aortitis.^12^ In addition, rapid deterioration, increased morbidity, and mortality could also occur if higher doses of glucocorticoids and biologic agents were initiated even if not indicated and if other secondary vasculitis aetiologies were not ruled out. We report a case series of three patients presenting with rare infectious pseudoaneurysm, infectious occlusive iliac thrombus, and paraneoplastic aortitis as vasculitis mimickers with poor, devastating outcomes.

## CASE 1

**Figure 1.**
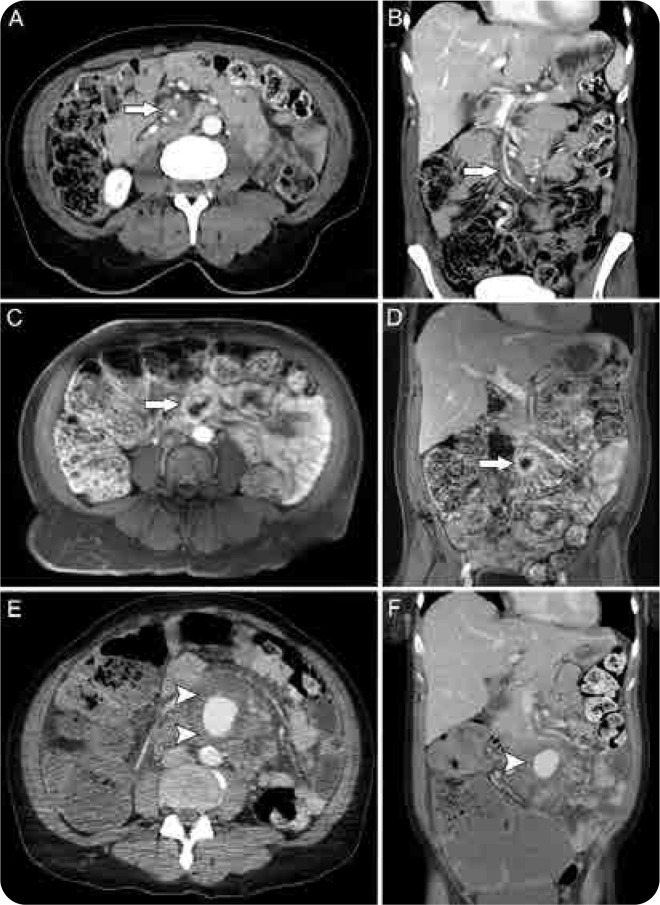
A composite figure of a mycotic aneurysm of the SMA (superior mesenteric artery) with a subsequent rupture. Contrast-enhanced axial (**A**) and coronal (**B**) CT images of the abdomen and pelvis show a significant soft tissue thickening enveloping the SMA, suspicious for a mycotic aneurysm (arrows). Subsequent same-day MRI/MRA study of the abdomen further confirmed the diagnosis and the same findings (arrows) can be seen on postcontrast sequences in axial (**C**) and coronal planes (**D**) respectively. A contrast-enhanced CT obtained two days later revealed interval aneurysm rupture (arrowheads) and developing haemoperitoneum, again seen on axial (**E**) and coronal images (**F**). Also seen is a markedly distended proximal colon on the coronal image (**F**).

A woman in her early 50s with a medical history of human immunodeficiency virus (HIV)/AIDS, hypertension, hidradenitis suppurativa, Crohn’s disease, cocaine and tobacco abuse presented with nausea, vomiting, and abdominal pain for the past 4 days. She was treated in the past for gonorrhoea and mycoplasma genitalium infection and had prior ileostomy for fistulas associated with her Crohn’s disease. She reported a history of bloody stools but denied skin rash, joint pain and swelling, chest pain, shortness of breath, focal neurologic deficits, and blood in sputum and urine. Upon admission, she was hypertensive at 165/91 mmHg and afebrile (98.4 °F) with a good oxygen saturation of more than 95%. The examination was notable for bruising on the right superior shoulder, macular pigmentation on the posterior thorax, multiple healed scars on axillary areas, abdomen, and lower extremities, and a distended abdomen with diffuse tenderness. Metronidazole was initiated. Laboratory revealed elevated sedimentation rate (ESR) (83 mm/hr) and high sensitivity c-reactive protein (CRP) (19.70 mg/L), haematuria, and mild proteinuria; negative antinuclear antibody (ANA), rheumatoid factor (RF), ANCA panel, PR3 and MPO antibodies, and complement levels. The infectious panel, including QuantiFERON TB Gold Plus, syphilis, and blood culture, was negative. HIV viral load was elevated, and the CD4 ratio was less than 200. Chest X-ray is unremarkable. Computed tomography (CT) of the abdomen and pelvis did not show evidence of small bowel ischemia or obstruction but were notable for fat stranding and enhancement surrounding a distal branch of the superior mesenteric artery (SMA) with mildly irregular lumen and mesenteric infiltration along mesenteric vessels concerning for vasculitis (**Figure 1**). Neck CT angiography (CTA) did not show evidence of large vessel occlusion, aneurysm, and segmental arterial mediolysis. Chest CT revealed centrilobular emphysema and bilateral small pulmonary nodules, the largest measuring up to 4.5 mm in the right upper lobe, with solid and ground glass complements. Magnetic Resonance Angiography (MRA) of the abdomen with and without contrast revealed luminal irregularities and increased enhancement of the posterior wall of the proximal SMA beginning just distal to the inferior pancreaticoduodenal artery branch and extending beyond the visualised jejunal branch with focal fusiform dilation of the arterial lumen at this level, with a diameter measuring approximately 8.4 mm. During hospitalisation, she remained on antibiotics but still developed worsening abdominal pain, spiking fevers, and non-bloody vaginal discharge. There were no signs or symptoms concerning systemic vasculitis, such as purpura or new neurological symptoms. Stat X-ray of abdomen/KUB was unremarkable for bowel perforation or obstruction. However, repeat abdominal and pelvic CT revealed severe enlargement of the distal SMA branch, likely middle colic resulting in mycotic pseudoaneurysm and likely rupture with contained bleed, large mesenteric hematoma surrounding the pseudo-aneurysm, and distended large bowel loops measuring up to 8 cm in diameter concerning for developing obstruction. She went into haemorrhagic shock and cardiac arrest with worsening abdominal distention and elevated intraabdominal pressures. Emergency exploratory laparotomy was done, revealing a massive hemoperitoneum, dilated bowel with adhesions between the omentum and pelvic structure, and mycotic aneurysm of SMA. She eventually expired with refractory haemorrhagic shock.

## CASE 2

**Figure 2.**
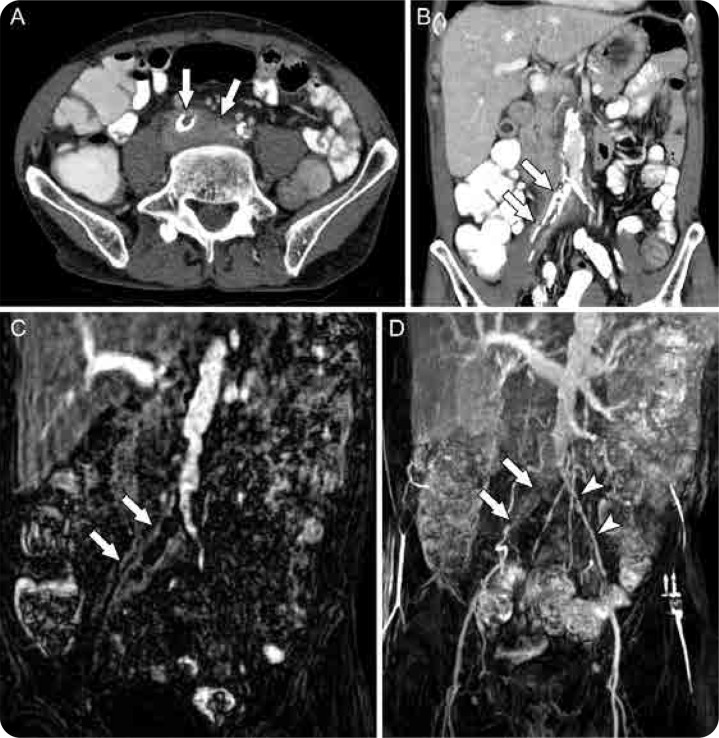
A composite figure showing vasculitis of the distal abdominal aorta and right iliac arteries with associated thrombosis. Contrast-enhanced CT of the abdomen and pelvis showed soft tissue thickening and thrombosis of the right common and external iliac arteries (arrows) as can be seen in the axial (**A**) and coronal (**B**) images. A coronal MRA image (**C**) also demonstrated a thrombus in the same vessels and surrounding soft tissue enhancement, further supporting the diagnosis of vasculitis (arrows). A coronal MIP (maximum intensity projection) MRA image (**D**) shows the occlusion of the right iliac arteries (arrows). Contralateral left common and external arteries are labelled for comparison (arrowheads).

An immigrant man in his mid-60s with a history of chronic alcohol and tobacco abuse and previously treated pulmonary tuberculosis presented with a week-long history of fevers, chills, and general weakness. SARSCoV-2 and influenza tests were negative. He reported chronic lower back pain and lower extremity numbness from a car accident two years ago. He denied any recent gastrointestinal infection. He worked docking boats and did not have pets or domestic animals. Upon admission, he was hypotensive (79/60 mmHg), tachycardic (120 bpm), and febrile (101.3°F). Laboratory showed no leucocytosis and bandaemia (bands 38%), mild lactic acidosis (2.4 mmol/L), with elevated hs-CRP (121.3 mg/L) and ESR (88 mm/hr). He was started on broad-spectrum antibiotics Vancomycin and Piperacillin-Tazobactam. Infectious panel, including influenza, respiratory syncytial virus, SARS-CoV-2, HIV, hepatitis B, C, and syphilis, was negative except for QuantiFERON TB Gold Plus. Chest X-ray was negative. Initial blood cultures showed Gram-negative rods. Transthoracic echocardiography indicated a normal ejection fraction of 55%, mild tricuspid regurgitation, and trace aortic regurgitation without valvular vegetations or abscesses. Non-contrast CT of the abdomen and pelvis showed circumferential bladder wall thickening, trace right perinephric and periureteral fat stranding, and a distended gallbladder with questionable mural thickening (**Figure 2**). The right upper quadrant ultrasound (US) was negative for obstruction. Lumbar spine MRI did not show osteomyelitis or discitis. Despite treatment, he continued to have fevers, and antibiotics were switched to Meropenem. A contrast CT of the abdomen and pelvis revealed ill-defined soft tissue surrounding the distal abdominal aorta and right common and external iliac arteries, suggesting thrombosis with presumed vasculitis. MRA of the chest and abdomen showed an occlusive thrombus extending from the origin of the right common iliac artery to the distal right external iliac artery, with retroperitoneal perivascular inflammation and soft tissue thickening extending from the distal abdominal aorta to the bifurcation of the right common iliac artery. Oral anticoagulation was started. On the seventh day of hospitalisation, *Campylobacter (C.) fetus* was identified. He improved clinically over two weeks and was discharged on Meropenem and three months of anticoagulation. However, despite multiple communication attempts, he was lost to follow-up after discharge.

## CASE 3

**Figure 3.**
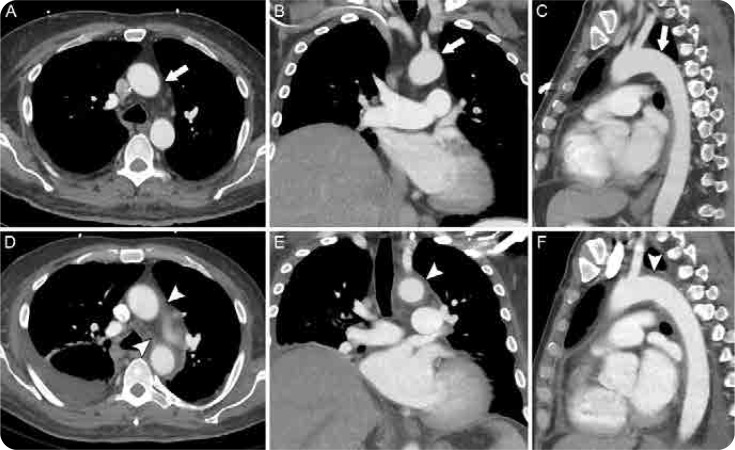
A composite figure of an aortitis. Axial (**A**), coronal (**B**) and sagittal (**C**) images of a CT chest with IV contrast approximately 3 months before presentation showing unremarkable mediastinum, specifically lack of fat stranding and thickening in the mediastinal fat (arrows). Axial (**D**), coronal (**E**) and sagittal (**F**) images of a CT chest with IV contrast obtained during the admission demonstrate mediastinal fat stranding and significant soft tissue thickening surrounding the thoracic aorta (arrowheads). Incidentally seen is moderate right pleural effusion (**A**).

A man in his mid-50s with a recently diagnosed acute myeloid leukaemia (AML) with myelodysplasia eight months ago and vancomycin-resistant enterococci (VRE) bacteraemia presented with severe anterior chest pain. He was refractory to his chemotherapy regimen, which was stopped three months ago, and was considered a stem cell transplant, which he declined. He reported his chest pain radiates to the posterior neck. He denied any shortness of breath, recent colds, cough, jaw or arm claudication, headaches, visual changes, or scalp tenderness but endorsed high-grade fevers to 103 °F. He was treated for colonic diverticulitis and perirectal abscess 3 months prior and a repeat CT of the abdomen and pelvis showed persistent versus recurrent diverticulitis. He was treated with Vancomycin, Metronidazole, and Cefepime. Examination showed hypoxia, mild bibasal crackles, and left leg swelling and redness. There were no murmurs or carotid bruit and pulses on extremities were present and symmetric. Laboratory showed severe neutropenia (500 cells/mm^3^), anaemia, and thrombocytopenia. Chest X-ray revealed nonspecific perihilar and basilar interstitial opacities. CT of the chest did not show any pulmonary emboli but revealed new acute inflammatory changes in the mediastinum which circumferentially involve the aortic arch, proximal arch vessels, and proximal descending aorta concerning for aortitis along with periaortic stranding in the thoracic aorta (**Figure 3**). Transthoracic echocardiogram demonstrated reduced left ventricular systolic function. Doppler US of the left lower extremity was negative for thrombosis. Infectious panel, including hepatitis, HIV, tuberculosis, syphilis, blood culture, and fungal culture, was negative. Autoimmune serologies, including IgG subclass levels, ANA, SSA, SSB, and complement levels, were negative. He continued to have spiking fevers and antibiotics were switched to Daptomycin and Meropenem while Posaconazole and Acyclovir were added. He also received multiple packed red blood cell and platelet transfusions. Despite treatment, he continued to deteriorate and was eventually discharged to inpatient hospice and expired.

## DISCUSSION

We presented a case series of aortic pseudoaneurysm, occlusive iliac thrombus, and aortitis related to infectious and paraneoplastic aetiologies. A high index of suspicion for other underlying aetiologies for secondary vasculitis such as infection and malignancy was considered based on the patients’ ages, immunocompromised status, comorbidity burden, and clinical presentations. Steroids and biological therapy were held until comprehensive investigations were completed. Identification of a wide spectrum of differential diagnoses for vasculitis could range from infectious, non-infectious, inflammatory, and non-inflammatory etiologies.^2^ It is crucial to rule out infectious and malignant causes for vasculitis mimickers and secondary vasculitis before diagnosing a primary vasculitis.^11^ This would lead to proper management and avoidance of unnecessary therapies that have significant adverse events and toxicities and have huge potential to worsen the disease.^13^ Recognising the characteristic clinical, serologic, diagnostic, and pathologic features for each aetiology is critical, especially with those atypical presentations not responding to the usual course and response with management for primary vasculitis. Identifying patients’ risk factors such as age, gender, medical and surgical comorbidities, environmental and nutritional status, and conditions predisposing to severe infections and their potential complications, will help establish the diagnosis (**Table 1**). In addition, recognising the onset of symptoms post-infection in the susceptible age groups could provide clues for infectious aetiologies for vasculitis such as HBV-PAN occurring less than a year after HBV exposure/infection and more in younger populations.^10^ Our patients presented with chronic immunosuppression from HIV/AIDS and other autoimmune diseases (Crohn’s disease and hidradenitis), chronic alcoholism with possible malnutrition status, and malignancy with recent chemotherapy immunosuppression causing them more susceptible to infectious aetiologies.

**Table 1.** Risk factors predisposing to infectious aortitis.^6,22^

Demographics Elderly Pregnant Medical and surgical history a. Chronic diseases Cardiovascular and renal diseases, atherosclerosis, uncontrolled diabetes mellitus, hypertension Congenital and preexisting vascular malformations History of aortoplasty, transplants, prostheses, iatrogenic intravascular procedures Level of Immunosuppression Co-existing malignancies and autoimmune diseases Immunosuppressive drugs and chemotherapy Low CD4 counts and other diseases with dysregulated cell-mediated immunity Radiation exposure Previous and current infection and reinfection Chronic nasal carriage (e.g., Staphylococcus aureus) HIV/AIDS Nutrition status Lifestyle/Recreational factors Intravenous drug use, smoking Environment High risk of pathogen exposure and endemicity

The most common pathogens for infectious aortitis were reported in several literatures (**Table 2**). Mycotic aortic aneurysms are rare with an incidence of 0.6–2% of all aortic aneurysms in Western populations.^14^ These infections do not involve fungal organisms or “mycosis” but are associated with bacterial endocarditis and infections with Group A β-haemolytic *streptococci* (GABHS), *pneumococci*, and *Haemophilus influenza.*^12^ An 18-year retrospective multicentre study of infectious aortitis was conducted in France based on blood culture, tissue sample from the aorta, and imaging changes.^15^ Majority of the reported cases were infectious aortitis secondary to Gram-positive microorganisms (44%), of which 50% are from streptococcus, while Gram-negative rods and intracellular growth and/or fastidious microorganisms comprise 33% and 43% of cases, respectively. In addition, most of the infectious aortitis were abdominal in location (56%); 22% of the patients were immunosuppressed either with immunosuppressive drugs, malignancy, or chronic infection. A literature review by Cox et al. also supported 60% of thoracic aortitis were secondary to Gram-positive microorganisms.^12^ With regards to location, most infectious vasculitis involves the para-visceral and thoracoabdominal aortas, especially the infrarenal aortas.^10,16^ On the other hand, tertiary syphilis involves the classic ascending aortitis involvement. In our first case, the mycotic aneurysm involved the superior mesenteric artery, which is the second major branch of the abdominal aorta. The patient’s blood cultures were negative; however, her worsening abdominal distention along with bowel dilatation suggestive of toxic megacolon could be the potential source for infection leading to this mycotic pseudoaneurysm. The second case revealed a *C. fetus* mycotic aneurysm, which involves a fastidious, Gram-negative bacteria that colonises the mucosal surfaces of the intestinal and reproductive tracts. It is a very rare aetiology but is reported to cause occlusive aortoiliac thrombus and aortitis with a mortality rate as high as 15–30% within 30 days.^17,18^

**Table 2.** Most common pathogens for infectious aortitis and mycotic aortic aneurysms.

**Type of pathogen**	**Study**
Salmonella, Staphylococcus aureus, Streptococcus pneumonia, Haemophilus influenza, Mycobacterium tuberculosis, HIV, and Treponema pallidum	Deipolyi et al., 2018^6^
Group A β-haemolytic *streptococci* (GABHS), *pneumococci*, and *Haemophilus influenza*	Cox et al., 2023^12^
*Streptococcus* species, *Staphylococcus aureus*, and *Salmonella* species	Sorelius et al., 2016^14^
Gram-positive microorganisms, particularly streptococcus, gram-negative rods and intracellular growth, and/or fastidious microorganisms	Journeau, et al., 2020^15^

With the advent of Coronavirus disease (COVID-19), several cases of aortitis were also reported.^19^ Symptoms were described as high fevers, generalised weakness, chest pain, back pain, vague abdominal pain, and weight loss. Physical examination was unremarkable except for abdominal tenderness in 25% of patients.^19^ Further workup includes detection of active acute infection by polymerase chain reaction (PCR) or evidence of previous COVID-19 infection after two months of acute symptom onset, persistent and markedly elevated CRP more than >100 mg/L, absence of COVID-19-related aortic thrombosis, and negative autoimmune and infectious panel. One case report showed elevated interleukin-6 levels in COVID aortitis.^20^ Involvement of aortitis as evidenced by abdominal CT/FDGPET also differed in acute and chronic COVID cases. Shorter segments of aorta involvement are observed in more acute COVID-19 cases compared to more diffuse aortitis in patients who have recovered from initial COVID-19 infection. Rare cases of transient aortitis related to COVID-19 vaccination were also reported and predominantly involved the ascending aorta and aortic arch.^21^

Paraneoplastic syndrome and hematologic malignancies are other aspects that must be ruled out with vasculitis mimickers.^13^ Erdheim–Chester disease is a non-Langerhans histiocytic disease and fibrosis that affects multiple organs, causing retroperitoneal infiltration and periarterial thickening of the aorta and its main branches. Cardiac myxomas can also present with vasculitis of any vessel size. Other malignancies such as intravascular large cell lymphoma could serve as a mimic of primary angiitis of the CNS. Another study mentioned aortitis associated with other occult malignancies, specifically solid tumours such as colon adenocarcinoma.^22^ In our third patient, his infection and autoimmune panels were negative in the setting of refractory AML despite chemotherapy. However, infection could not be completely ruled out given his immunosuppression status and history of recent multiple infections, including VRE bacteraemia, persistent colonic diverticulitis, and perirectal abscess 3 months ago.

### Physical examination

Considering the treatment approach is different based on underlying aetiology and pathology, awareness of red flags using comprehensive history and physical examination is imperative. Identification of more localising physical symptoms or signs pointing to infectious aetiologies could involve Osler’s nodes and Janeway lesions in endocarditis, internal jugular phlebitis in *Fusobacterium* pharyngitis, and ecthyma gangrenosum and more necrotising and destructive pulmonary haemorrhages with *Pseudomonas aeruginosa* infection.^10^

### Laboratory and pathologic workup

Signs of subacute to chronic inflammation observed in primary vasculitis could be found in patients with infectious aortitis.^11^ This includes leucocytosis, mild to moderate thrombocytosis, normochromic/normocytic anaemia, and elevated inflammatory markers. Other indicators of systemic inflammation such as hypoalbuminemia and polyclonal hypergammaglobulinemia could also give clues to vasculitis syndromes.

Histopathological results through biopsy of the involved vessel or tissue are important in confirming diagnosis, although may not be accessible and practical in some cases due to the huge risk for biopsy complications.^6^ Tissue biopsy will reveal neutrophilic infiltration of the arterial wall as a response to acute inflammation leading to the saccular appearance in imaging. Body fluid and tissue culture, staining, and sensitivity along with other microbiological testing, molecular analyses, detection of antigens and antibodies, and serologies for testing Bartonella, hepatitis, HIV, Lyme disease, syphilis, varicella-zoster virus, tuberculosis, fungal infections, and SARS-CoV-2, should be done to rule out infectious pathology first and could even take months for final microbiology reports.^23^ Maningding et al. also highlighted that these infectious aetiologies could also produce antibodies including positive ANCA, MPO, and PR3 antibodies such as found in infective endocarditis, and could complicate further in establishing primary versus secondary vasculitis.^23^

### Imaging studies

Diagnostic imaging tests for vasculitis include conventional angiography, MRA, and CTA, which were utilised in our cases, colour Doppler ultrasonography (CDU), and positron emission tomography (PET) with 18F-fluorodeoxyglucose (18F-FDG-PET).^24^ CT and MRI are essential for detailed measurement of the external vessel wall and diameter.^25^ The more invasive CT angiography might impose greater risks on highly fragile and acutely inflamed walls, but constitutes as the best high-resolution three-dimensional diagnostic imaging for more detailed vascular anatomy.^26^ This is required in presurgical planning and prognostication for impending new or rapidly growing aneurysms and ruptures in aortitis. Kesser et al. suggested PET/CT for monitoring disease activity of vasculitis and excluding malignancy along with hybrid PET/MRI due to lesser ionising radiation exposure.^2^ Imaging findings suggestive of infective aortitis involve thickened wall with loss of contour.^26^ Findings such as saccular appearance of the aorta, peri-aortic inflammatory infiltrate and oedema, gas in the aortic wall along with fat stranding represent infected aneurysms with higher risk for rupture.^12^ True non-infected aortic aneurysms on the other hand involve more fusiform appearance on imaging.^6^ In general, pseudoaneurysms are very common complications of infectious vasculitis although true aortic aneurysms and stenosis are also reported.^13^ This supports the importance of early imaging as a crucial diagnostic modality in investigating and comparing infected versus non-infected aneurysms, especially with a high index of clinical suspicion.^12^ Transoesophageal echocardiography is also necessary for infectious aortitis workup and the gold standard for ruling out infective endocarditis.^27^

### Treatment

Treatment of infectious vasculitis including mycotic involvement of the aorta and its branches must be emphasised before initiation of immunosuppressant and disease-modifying agents. Effective antimicrobial therapy such as empiric and combination bactericidal/bacteriostatic agents covering most common organisms of Gram-positive cocci and Gram-negative rods should be instituted early.^14^ Ampicillin or cephalosporins are better choices for Salmonella coverage, which is associated with higher mortality and for high risk patients with a delay to blood culture results.^12^ Anti-fungal or anti-tuberculous therapy usually requires longer courses of treatment duration.^28^ Dose escalation and adjustments in narrowing antibiotic coverage depend on the specific pathogen from blood or perioperative tissue specimens, even though only 50% to 82% are reported with positive blood cultures.^26^ Other factors for determining duration of antibiotics lie in the location, characteristics, and size of the lesion, along with vascular complications and the overall immunologic status of the patients.^26^ Supportive systemic treatment such as in cases of hemodynamic instability should also be implemented.^8^

In cases of COVID-related aortitis, excellent recovery within 2 weeks was observed after high-dose steroids of Prednisone 40 mg–60 mg daily for 4 weeks followed with a gradual taper and non-steroidal anti-inflammatory drugs (NSAIDs).^29^ Shimada et al. described a case of COVID-induced aortitis with spontaneous resolution even without NSAIDs or steroids.^30^

### Utility of surgery

Acute infection and inflammation should be resolved first before contemplating surgical interventions due to associated weakened aortic walls and increased risk of rupture in acute infectious aortitis.^24^ Surgery is indicated with rapid expansion of aneurysm or with aneurysm diameter greater than 5 mm on diagnostic imaging.^25^ Multiple surgical approaches are available depending on the patient’s individualised factors and the provider’s level of expertise. These could range from in situ to extra-anatomic reconstructions with open repairs as gold standard for Type IA aortic aneurysms involving the ascending aorta to antibiotic bound and silver-bound grafts, cryo-preserved allografts, and endovascular stenting.^12,25^ On the other hand, one systematic review highlighted the importance of immediate surgical referral upon diagnosis of mycotic aortic aneurysms.^31^ A large cohort study from 2000 and 2016 in Sweden reported thoracic endovascular aneurysm repair (TEVAR) as the primary intervention for mycotic thoracic aneurysms. Another nationwide study in Sweden also reported EVAR as the preferred treatment for mycotic abdominal aortic aneurysms.^32^ EVAR was found to have acceptable short and long-term survival although with high infection-related complications requiring long-term antibiotics for at least 6–8 weeks especially with high risk and critically ill patients.^25^ In the study of Journeau et al., 85% of the infectious aortitis cases underwent surgical interventions, with one-third of the patients dying within 1 month, regardless of the infectious etiology or location of aortitis.^15^ Severe sepsis and graft infections, aneurysm rupture and perforation, and postsurgical complications were observed in these cases. However, Cox et al. mentioned that greater mortality is still associated with increased risk of aortic rupture without surgical management in infective aortitis.^12^ Poorer outcomes are dependent on multiple independent factors before surgery such as older age, level of infection and virulence of pathogen, and location of aneurysm (e.g. suprarenal).^16^

### Prognosis

Mycotic aneurysms have an increased risk of morbidity and mortality associated with fatal vascular events such as rupture. Gram-negative bacilli, particularly non-typhoid Salmonella and Staphylococcus aureus, were observed to have the worst outcomes and mortalities with their microbial invasion to the vessel intima leading to supra-renal aneurysms and ruptures.^12^ Pericardial effusion and myocardial infarction were also associated with mycotic coronary aneurysms. Study by Oderich et al. revealed an increased risk of mortality secondary to aneurysms due to the following risk factors: female sex, extensive periaortic infection, *Staphylococcus aureus* bacteraemia, rupture, and suprarenal location.^16^ Septic shock and its complications along with multiorgan involvement and perinatal infections could be induced by different pathologic mechanisms including direct vascular invasion and damage, direct seeding, and autoantibody production as part of infection-triggered immune dysregulation.^33^ In cases of COVID-related aortitis, the prognosis was favourable as evident by improved clinical response, decrease in acute phase reactants, and partial to complete disease resolution on repeat imaging.^19^

Combination of earlier diagnosis, surgical interventions, and targeted microbial therapy has resulted in tremendous improvements in outcomes for infectious aortitis.^12^ On the other hand, treatment of the underlying primary malignancy such as surgical excision, chemotherapy, or radiotherapy could have a dramatic improvement in symptoms, disease activity, and inflammation for paraneoplastic aortitis.^34^ Thus, these interventions should be done along with potentially managing secondary vasculitis and aortitis with corticosteroids if continued to have no spontaneous improvement and recovery with the above interventions. Return of constitutional symptoms after a period of remission could indicate cancer recurrence, therefore close surveillance is crucial. In addition, once infection and malignancy are ruled out, other cases might need treatment for inflammatory vasculitis such as biologic therapy especially if clinical and diagnostic presentations are not self-limiting and progressive. In our cases, none of the patients received glucocorticoids or immunosuppressive agents. Risks for severe opportunistic infections were reported in several studies, especially within the first year of the treatment course of vasculitis, not to mention associated drug toxicities and side effects.^35^

## CONCLUSION

This case series emphasises the critical need for prompt clinical recognition of infectious and paraneoplastic aetiologies as part of differential diagnosis for aortitis, which poses diagnostic challenges, life-threatening vascular complications, and higher mortality if missed and undetected. Individualised therapy must be based on the appropriate and extensive diagnostic workup, including utilisation of diagnostic imaging, microbiologic studies, and histopathologic findings, while delaying unnecessary use of glucocorticoids or immunosuppressive agents until secondary vasculitis is ruled out. A multidisciplinary collaboration among infectious disease specialists, rheumatologists, pathologists, vascular surgeons, and critical care intensivists, along with the patients and families, cannot be ignored in weighing the risks and benefits of every intervention and improving overall patient outcomes.

## AUTHOR CONTRIBUTIONS

Conceptualisation, comprehensive literature review C.A. and G.K.; writing—original draft preparation, C.A.; writing—manuscript review and revision and proof reading, editing, G.K. and C.A; provided images and legends, T.M. All authors are in agreement to be accountable for all aspects of the work in ensuring that questions related to the accuracy or integrity of any part of the work are appropriately investigated and resolved.

## DISCLAIMER

No part of this manuscript, including the text and graphics, are recycled, copied, or published elsewhere in whole or in part.

## CONFLICT OF INTEREST

The authors declare no conflict of interest.

## FUNDING

The authors did not receive a grant specifically for the research from any funding agency in the public, commercial, or not-for-profit sector.

## ETHICS STATEMENTS

This work obtained a waiver of approval from the Institutional Review Board of University of Florida.

## PATIENT CONSENT FOR PUBLICATION

Consent was waived according to compliance with the University of Florida IRB and Privacy rules for case reports.
